# Unilateral Syphilitic Nodular Scleritis: A Case Report

**DOI:** 10.7759/cureus.107714

**Published:** 2026-04-25

**Authors:** Meryem Benchekroun, Saad Benchekroun, Narjisse Taouri, Lalla Ouafa Cherkaoui

**Affiliations:** 1 Ophthalmology, Specialty Hospital of Rabat, Mohamed V University, Rabat, MAR

**Keywords:** anterior nodular scleritis, cutaneous syphilis, ocular syphilis, penicillin g, syphilis

## Abstract

Syphilis is a systemic infectious disease known as the “Great Masquerader” for its ability to mimic a wide range of ocular conditions, potentially involving any ocular structure. We report the case of a 26-year-old man with a history of unprotected sexual intercourse who presented with a two-week history of a painful, red right eye without loss of visual acuity. Examination revealed nodular lesions on the temporal sclera and concomitant reddish-brown palmar spots. Laboratory tests were performed to determine the etiology of the anterior nodular scleritis. Serologic testing (treponema pallidum hemagglutination assay (TPHA)/Venereal Disease Research Laboratory test (VDRL)) was positive, confirming syphilitic nodular scleritis. The patient showed rapid clinical improvement following penicillin therapy.

## Introduction

Syphilis is a systemic infectious disease, often referred to as the “Great Masquerader” because of its ability to mimic a wide range of ocular conditions [1]. Ocular involvement can affect nearly any structure of the eye and most commonly presents as uveitis. In contrast, scleral involvement is rare, accounting for approximately 3% of ocular syphilis cases [2].

Within this spectrum, syphilitic anterior nodular scleritis is a rare presentation. Its clinical features often overlap with those of other inflammatory scleral diseases, which can make the diagnosis challenging and lead to delays in appropriate management. Awareness of this rare entity is essential to ensure prompt recognition and appropriate management. Notably, syphilitic scleritis typically shows a poor response to corticosteroid therapy, whereas it demonstrates a favorable response to penicillin treatment [3].

## Case presentation

We report the case of a 26-year-old male prisoner who presented to the ophthalmology emergency department with a painful, red right eye for two weeks without a decrease in visual acuity. The patient reported having no particular medical history or general symptoms apart from a non-itchy rash on the palms of his hands. Furthermore, the patient reported a history of unprotected sexual intercourse.

**Figure 1:**
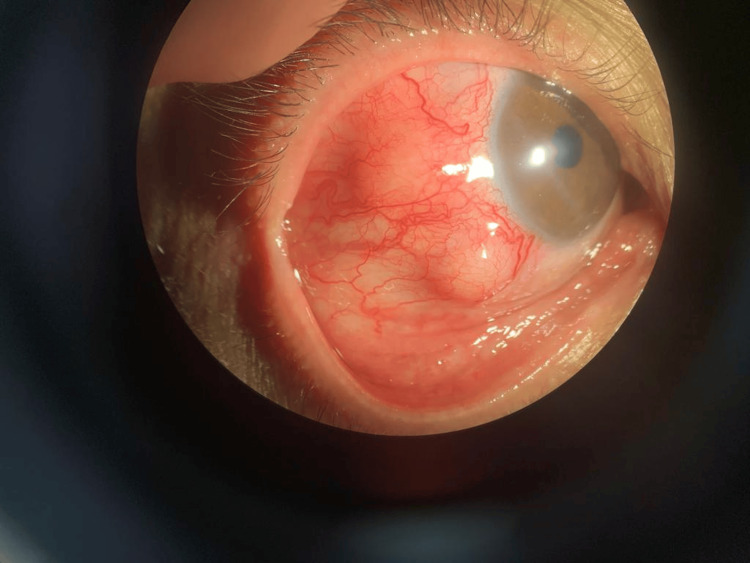
Right eye showing anterior nodular scleritis

The clinical examination found a visual acuity of 20/20 in both eyes. Slit-lamp examination found two solid, injected, nonmobile nodular lesions measuring 4 mm each located on the temporal sclera of the right eye (Figure 1).

**Figure 2:**
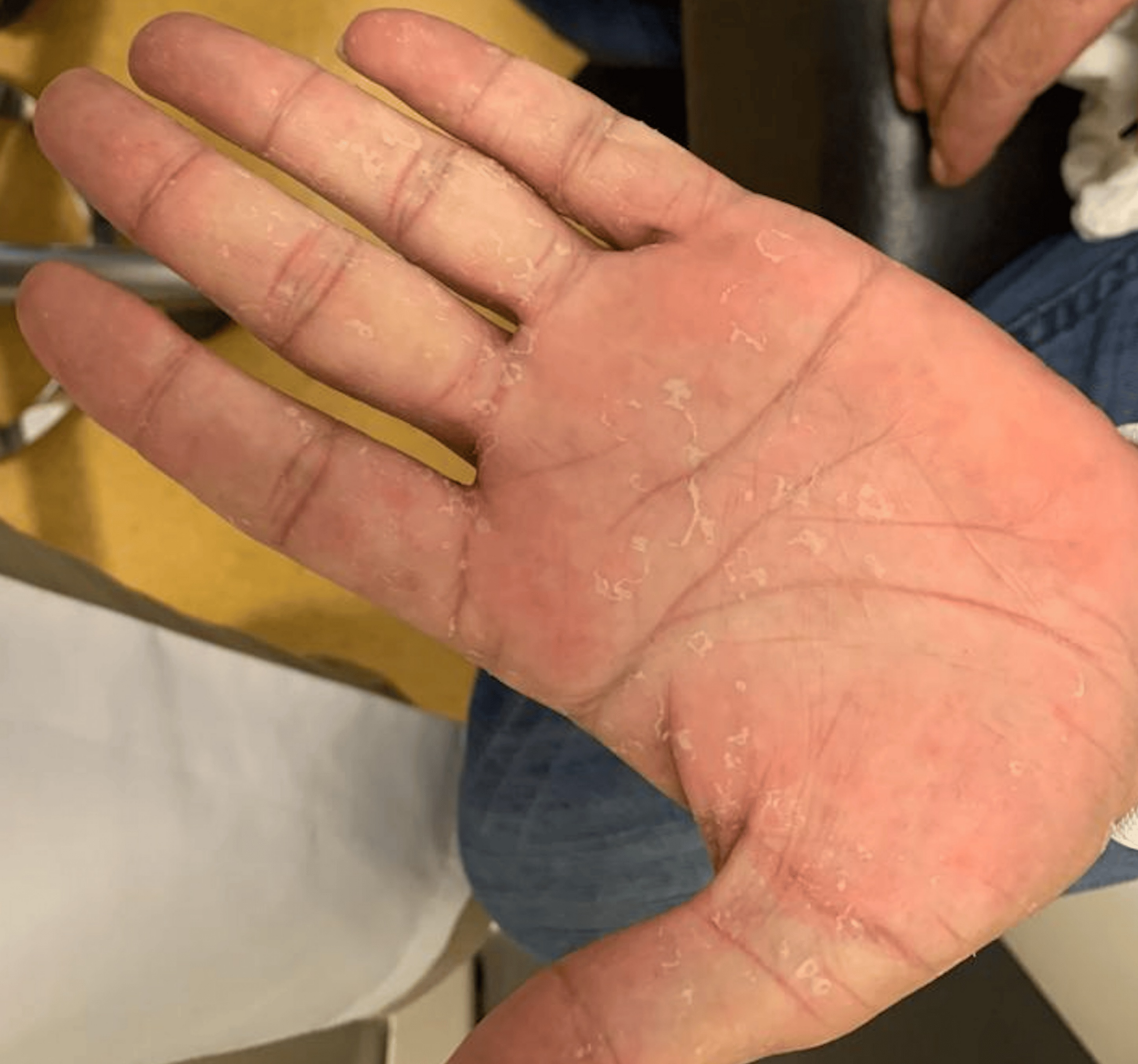
Hand of the patient showing palmar lesions

The rest of the examination of both eyes was strictly normal. The intraocular pressure was 10 mmHg for the right eye and 12 mmHg for the left eye, with a deep and quiet anterior chamber and a normal fundoscopic examination in both eyes. On the palms of the patient’s hands, reddish-brown spots were observed (Figure 2).

Laboratory tests were performed to determine the etiology of the nodular scleritis. These tests included complete blood count, angiotensin-converting enzyme, QuantiFERON test for tuberculosis, Treponema pallidum antibodies, and rheumatoid factor. A chest radiograph was also performed. All the results came back negative except for the treponema pallidum hemagglutination assay (TPHA)/Venereal Disease Research Laboratory test (VDRL), which came back positive. The patient was then referred to a neurologist for a neurological examination and a lumbar puncture, the result of which was normal (Table 1).

**Table 1:** Findings of the laboratory investigations of the patient CSF, cerebrospinal fluid; TPHA, treponema pallidum hemagglutination assay; VDRL, Venereal Disease Research Laboratory test

Parameters	Patient values	Reference range
Hemoglobin	14 g/dL	13-17 g/dL
White blood cell count	5500 cells per microliter	4000-11000 cells per microliter
Platelet count	250000 platelets per microliter	150000-450000 platelets per microliter
Angiotensin-converting enzyme	25 U/L	20-70 U/L
QuantiFERON	0.1 U/mL	≤0.35 U/mL
TPHA	Positive	Negative
VDRL	1:64 (positive)	Negative
Rheumatoid factor	5 U/mL	<20 U/mL
Appearance of the CSF	Clear	Clear
Opening pressure	14 cmH₂O	10-20 cmH₂O
White blood cells (CSF)	2 cells/mm³	0-5 cells/mm³
Glucose (CSF)	2.8 mmol/L	2.5-4.5 mmol/L
Protein (CSF)	0.35 g/L	0.15-0.45 g/L

**Figure 3:**
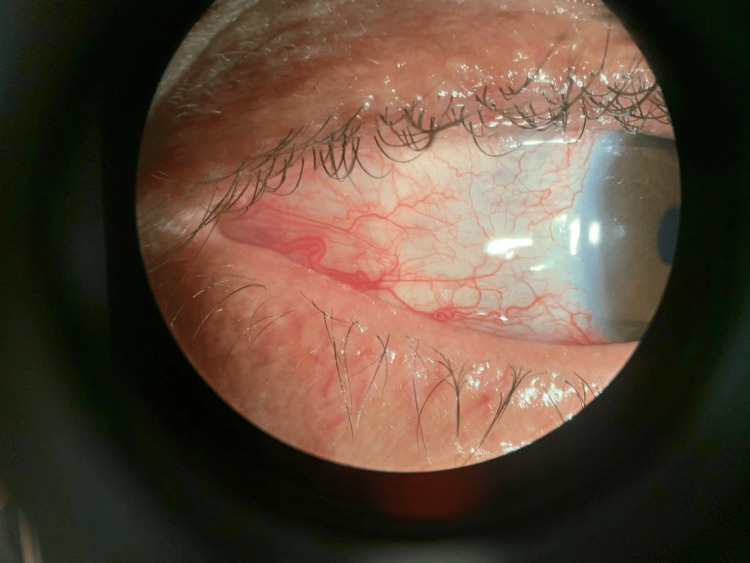
Right eye showing the evolution of the scleritis after treatment

Aqueous crystalline penicillin G was administered intravenously to the patient at a dose of 18-24 million units per day for 14 days. The clinical course was marked by a rapid improvement of the lesions within 10 days (Figure 3). In addition, serological testing for human immunodeficiency virus (HIV) and hepatitis B and C was performed and returned negative results.

## Discussion

Syphilis is a systemic infectious disease caused by the spirochete Treponema pallidum, primarily transmitted through sexual contact with infectious lesions [1]. Owing to its hematogenous dissemination, the disease can involve multiple ocular structures, including the conjunctiva, cornea, sclera, and optic nerve. Consequently, ocular syphilis presents with a wide spectrum of manifestations, such as interstitial keratitis, uveitis, and papillitis [3].

Among these manifestations, syphilitic scleritis remains a rare entity, accounting for approximately 3% of ocular syphilis cases. It most frequently presents as anterior nodular scleritis [2,4]. The rarity of this condition, combined with its nonspecific clinical presentation, contributes to diagnostic challenges.

Syphilis is often referred to as “the great imitator” due to its ability to mimic a variety of other diseases. In the context of anterior nodular scleritis, several differential diagnoses must be considered, including infectious and autoimmune conditions such as tuberculosis, giant cell arteritis, sarcoidosis, and rheumatoid arthritis [5]. This overlap in clinical features underscores the importance of thorough etiological investigation.

From a therapeutic perspective, syphilitic scleritis typically demonstrates a poor or limited response to corticosteroid therapy when used alone. In contrast, it responds favorably to appropriate antibiotic treatment. The standard regimen for ocular syphilis consists of aqueous crystalline penicillin G administered intravenously at a dose of 18-24 million units per day for 10 to 14 days. Alternatively, intramuscular procaine penicillin (2.4 million units daily) may be used over the same duration [6].

Consistent with the literature, previously reported cases of syphilitic nodular scleritis have shown favorable outcomes following antibiotic therapy, with resolution of scleral nodules generally observed within one to two weeks after initiation of treatment [2,4]. This rapid clinical improvement further supports the infectious etiology and highlights the importance of early diagnosis and targeted management.

## Conclusions

Even though syphilitic scleritis is rare, it should be suspected and ruled out in all patients with scleritis for rapid diagnosis and treatment, especially since syphilitic scleritis responds very well to penicillin therapy.

Furthermore, it is important to educate the general public about the prevention of syphilis and other sexually transmitted diseases as part of the primary prevention of these diseases and to promote the use of condoms.
